# Targeting myeloid-derived suppressor cells to enhance natural killer cell-based immunotherapy

**DOI:** 10.1016/j.pharmthera.2022.108114

**Published:** 2022-02-02

**Authors:** Shweta Joshi, Andrew Sharabi

**Affiliations:** a Department of Pediatrics, Division of Pediatric Hematology-Oncology, Moores Cancer Center, University of California San Diego, La Jolla, CA 92093-0815, USA; b Moores Cancer Center, Department of Radiation Medicine and Applied Sciences, University of California, San Diego, La Jolla, CA, USA

**Keywords:** myeloid-derived suppressor cells, natural killer cells, T cells, tumor microenvironment, immunotherapy

## Abstract

Myeloid-derived suppressor cells (MDSCs) are immune cells of the myeloid lineage that progressively accumulate in tumors and play an important role in promoting tumor growth. MDSCs interact with other immune cells present in the tumor microenvironment (TME) and utilize multiple mechanisms to promote immunosuppression. On the other hand, natural killer (NK) cells are cytotoxic cells of the innate immune system and work as one of the first lines of defense against tumors. However, the role of MDSCs in regulating or suppressing NK cells within the TME is poorly understood. This review discusses MDSC-associated immunosuppression, the mechanisms regulating communication between MDSCs and NK cells in the tumor microenvironment, and how MDSC may impact NK-cell-based immunotherapies. We also explore various strategies to increase NK cell cytotoxicity by blocking MDSC-mediated immunosuppression with the goal of enhancing cell based anticancer therapeutics.

## 1. Introduction

The tumor microenvironment (TME) is a complicated ecosystem made up of proliferating tumor cells, stromal cells, immune cells, and non-cellular components which interact and crosstalk in different ways to determine tumor progression and clinical outcome (Bejarano, Jordao, & Joyce, 2021; Binnewies et al., 2018; Hinshaw & Shevde, 2019). It is now clearly established that infiltrating immune cells are present in all solid tumors from the initial stages of tumor development (Grivennikov, Greten, & Karin, 2010) and play a key role in controlling tumor growth and determining responses to therapies (Fridman, Pages, Sautes-Fridman, & Galon, 2012; Holzel, Bovier, & Tuting, 2013; Palucka & Coussens, 2016). During the development of neoplasia, natural killer (NK) cells and T cells are thought to be the predominant infiltrating cell types; however, as tumors progress these cells are outnumbered by regulatory T cells (Treg), myeloid-derived suppressor cells (MDSCs), and tumor-associated macrophages (TAMs) which results in immunosuppression in the TME (Gonzalez, Hagerling, & Werb, 2018; Joshi, 2020). The potency of effector immune cells, namely CD8+ T cells and NK cells, to identify and eliminate tumor targets is influenced by the presence of cells of the myeloid lineage, namely MDSCs and TAMs in the TME, which utilize several strategies to turn off the tumor-killing functions of NK cells and T cells (Ostrand-Rosenberg, Sinha, Beury, & Clements, 2012; Uzhachenko & Shanker, 2019). In other words, MDSCs directly interact with T cells and NK cells to induce an immunosuppressive tumor microenvironment and promote tumor growth (Bruno, Mortara, Baci, Noonan, & Albini, 2019; Fleming et al., 2018; Groth et al., 2019; Joshi & Durden, 2019; Law, Valdes-Mora, & Gallego-Ortega, 2020; Zalfa & Paust, 2021). While the molecular mechanisms by which MDSCs inhibit the functions of T cells are widely studied (Draghiciu, Lubbers, Nijman, & Daemen, 2015; Fultang et al., 2019; Groth et al., 2019), the impact of MDSCs on the function of NK cells has not been explored in as much detail. Here we review the phenotypes and functions of MDSCs and NK cells and discuss the crosstalk/communication between MDSCs and NK cells, NK-cell-based immunotherapies, and strategies to target MDSCs to enhance NK cell cytotoxicity.

## 2. MDSC: Origin, development, and subsets

**Fig. 1.**
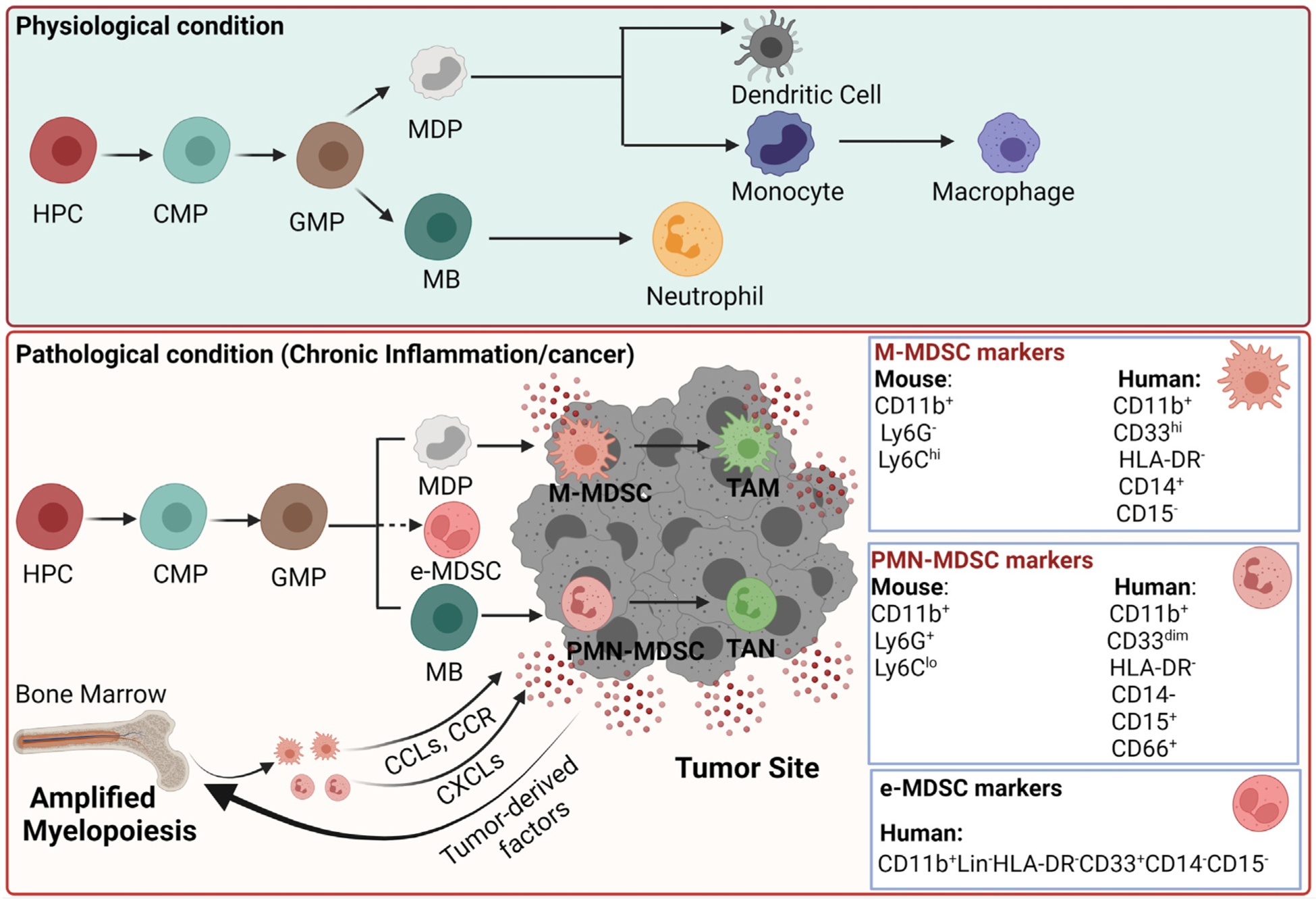
Differentiation of myeloid-derived suppressor cells in the TME. Under physiological conditions, hematopoietic progenitor cells (HPC) in the bone marrow give rise to macrophages, dendritic cells, and neutrophils. HPC differentiates into granulocyte-macrophage progenitor (GMP) after common myeloid progenitor (CMP), and then monocytic/dendritic cell precursor (MDP) and myeloblasts (MB) arise from GMP. MDP differentiates into monocytes/macrophages and dendritic cells, and MB differentiates into neutrophils. However, under pathological conditions like cancer, myeloid cells expand and differentiate into monocytic MDSCs or polymorphonuclear or granulocytic (PMN-MDSCs or G-MDSCs). MDSCs are recruited to the tumors by the same chemokines that are used in the migration of monocytes and neutrophils. In the TME, M-MDSCs differentiate into tumor-associated macrophages (TAMs) and PMN-MDSCs into tumor-associated neutrophils (TANs). The fig. is created with Biorender.com.

In physiological conditions, common myeloid progenitors (CMP) are differentiated from hematopoietic progenitor cells (HPC) in the bone marrow (BM) (Fig. 1). Later CMP migrate to secondary lymphoid organs and differentiate into monocytes or neutrophils (Tcyganov, Mastio, Chen, & Gabrilovich, 2018). This pathway involves granulocyte-macrophage progenitors (GMP), myeloblasts (MB), and monocytic/dendritic cell precursors (MDP). In pathological conditions such as prolonged inflammation or cancer, alternative myelopoiesis augments the production of myeloid cells in the BM to combat these threats (Ribechini, Greifenberg, Sandwick, & Lutz, 2010; Takizawa, Boettcher, & Manz, 2012; Tcyganov et al., 2018). Various pro-inflammatory cytokines such as prostaglandin E2 (PGE2), granulocyte-colony stimulating factor (GCSF), granulocyte-macrophage stimulating factor (GMCSF), vascular endothelial growth factor (VEGF), transforming growth factor (TGFβ), and S100 proteins are released in the TME, which induces the recruitment of immature myeloid cells (IMC) to the tumor site and perturb the maturation of myeloid cells (Gabrilovich, Ostrand-Rosenberg, & Bronte, 2012). This process of myelopoiesis leads to a spectrum of immature myeloid cells, which are morphologically similar to monocytes or granulocytes but can be distinguished by the presence of specific cell surface markers (Ostrand-Rosenberg & Sinha, 2009). It is generally accepted that MDSCs are of mainly two types: monocytic MDSCs (M-MDSCs) and polymorphonuclear or granulocytic MDSCs (PMN-MDSCs or G-MDSCs) (Bronte et al., 2016; Umansky, Blattner, Gebhardt, & Utikal, 2016). Murine MDSCs were initially defined as cells expressing both CD11b and Gr1 surface markers (Bronte et al., 2016). This limited definition of MDSCs has been improved upon and murine MDSC subsets are further classified based on the surface expression of Ly6C and Ly6G. M-MDSCs are mainly characterized in mice by the expression of CD11b^+^Ly6C^hi^Ly6G^−^ surface markers, and PMN-MDSCs are described as CD11b^+^Ly6C^lo^Ly6G^+^. In humans, M-MDSCs are described as CD11b^+^CD33^hi^HLA-DR^−^CD14^+^CD15^−^ and PMN-MDSCs are characterized by expression of CD11b^+^CD33^dim^HLA-DR^−^CD14^−^CD15^+^CD66b^+^ (Fig. 1). Some recent studies have suggested that M-MDSCs display high expression of myeloid marker CD33 compared to PMN-MDSCs (Bronte et al., 2016; Veglia, Sanseviero, & Gabrilovich, 2021). Hence, in place of CD11b, CD33 can be considered an additional marker to differentiate human M-MDSCs from PMN-MDSCs. Additionally, M-MDSCs can be distinguished from monocytes based on the expression of HLA-DR (Bronte et al., 2016) and PMN-MDSCs can be distinguished from neutrophils based on the density gradient: PMN-MDSCs are seperated on a low-density gradient, while neutrophils are seperated on a high-density gradient (Bronte et al., 2016). In humans, these surface markers and strategies can be used to distinguish monocytic and granulocytic MDSC subsets from monocytes, neutrophils, and dendritic cells (Gabrilovich, 2017). However, in mice, no specific cell surface markers are available to distinguish classical monocytes from M-MDSCs and classical neutrophils from PMN-MDSCs. Hence, the same phenotypic markers that are used to identify monocytes and granulocytes are used for the recognition of M-MDSCs and G-MDSCs in mice, and they can be distinguished only on the basis of their potency to suppress the function of other immune cells (Veglia et al., 2021).

Recently, transcriptomic, and proteomic analysis has provided specific gene expression patterns to characterize different myeloid phenotypes in the TME (Choksawangkarn et al., 2016; Condamine, Mastio, & Gabrilovich, 2015; Gato et al., 2016). Several studies have shown that M-MDSCs and PMN-MDSCs can be discriminated from tumor-associated macrophages (TAMs) and tumor-associated neutrophils (TANs), respectively, based on the expression of specific cell markers (Bronte et al., 2016; Condamine et al., 2016; Dumitru, Moses, Trellakis, Lang, & Brandau, 2012). For example, M-MDSCs and TAMs can be distinguished based on low or medium expression of Ly6C, low or no expression of S100A9, and high expression of macrophage colony-stimulating factor (MCSF), F4/80, IRF8, and CSF1R in TAMs (Strauss et al., 2015). Similarly, PMN-MDSCs and TANs can be distinguished based on the recently discovered novel marker, lectin-type oxidized LDL receptor-1 (LOX-1), highly expressed in PMN-MDSCs isolated from peripheral blood of cancer patients (Condamine et al., 2016). In addition, the mRNA profile of TANs significantly differs from the profile of splenic neutrophils and PMN-MDSCs (Fridlender et al., 2012; Gabrilovich, 2017). A study by Fridlender et al has shown that splenic neutrophils show higher expression of structural genes and genes related to cell cytotoxicity and respiratory burst and the higher expression of these genes contributes to the anti-bacterial function of neutrophils (Fridlender et al., 2012). However, PMN-MDSCs show progressive loss of these pathways, and TANs show a dramatic loss or complete absence of these pathways. In contrast, many immune-related genes were over-expressed in PMN-MDSCs and were further expressed high in TANs. Song *et al* has performed single-cell RNA-seq to study the transcriptomes of tumor tissues and surrounding normal tissues isolated from patients of NSCLC (Song et al., 2019). This study has shown that M-MDSCs are molecularly distinct from M1 or M2-like TAMs. Taken together, these emerging studies have shown that specific markers or gene expression profiles can be used to delineate different myeloid populations, and these findings will aid strategic design of MDSC-targeted therapies in the near future.

In addition to M-MDSC and PMN-MDSC, another population of MDSCs termed early-stage MDSC (eMDSC) was recently characterized in humans. This phenotype constitutes only 3% of the MDSC population, and is likely composed of immature progenitor cells, and is characterized as CD11b^+^Lin^−^HLA-DR^−^CD33^+^CD14^−^CD15^−^ (Bronte et al., 2016; Gabrilovich, 2017; M. Jiang et al., 2020). The origin and functions of e-MDSCs are poorly understood. Besides, e-MDSCs, a unique population of fibrocytic MDSCs has also been described in humans. This population is represented as CD11b^+^HLA-DR^+^ and is found to be abnormally expanded in patients with pediatric sarcoma (H. Zhang et al., 2013). Like fibrocytes, this myeloid population expresses smooth muscle actin and collagen. Very few studies have reported the presence of this population in cancer patients (Gunaydin, Kesikli, & Guc, 2015; Mu et al., 2021; H. Zhang et al., 2013), and further studies are needed to understand the role of this population in the progression of cancer.

Interestingly, the frequency of circulating MDSCs in the peripheral blood (PB) of cancer patients has been linked with the progression of the disease, and hence it is considered a prognostic determinant of disease in various cancers (Diaz-Montero et al., 2009; Messmer, Netherby, Banik, & Abrams, 2015; Solito et al., 2014). Specifically, MDSCs have been described as a predictive marker in gastric cancer, esophageal cancer, pancreatic cancer (Gabitass, Annels, Stocken, Pandha, & Middleton, 2011), melanoma (Sade-Feldman et al., 2016), colorectal cancer (Shirasuna et al., 2020), non-small cell lung cancer (Vetsika et al., 2014), and breast cancer (Safarzadeh et al., 2019). The favored expansion of a particular subtype of MDSCs depends on various factors present in the TME. Hence the ratio of M-MDSCs and PMN-MDSCs infiltrated in the tumors differs with tumor type and the advancement of the disease (Liang, Lu, Zhao, & Lu, 2019; Solito et al., 2014). The presence of circulating e-MDSCs is reported in patients with head and neck cancer or ovarian cancer, but the frequency of e-MDSCs does not correlate with survival (Cassetta et al., 2020; Okla et al., 2019). In addition, e-MDSCs isolated from these patients showed no or very little suppression of T cell proliferation. Lv et al have recently shown that the frequency of e-MDSCs is high in the peripheral blood of patients who have acute myeloid leukemia (AML) (Lv et al., 2021). Further studies are needed to determine whether e-MDSCs are as important as M-MDSCs and PMN-MDSCs in serving as a prognostic marker in cancer and predicting responses to immunotherapy in cancer.

## 3. Molecular mechanisms regulating recruitment, expansion, and pro-tumorigenic activation of MDSCs

MDSCs are rarely observed under physiologic conditions, but MDSCs gradually accumulate in the BM, spleen, blood, lymph nodes, and tumors of cancer patients. MDSCs are also reported to expand in initial and advanced cancer stages in preclinical mouse models (Diaz-Montero, Finke, & Montero, 2014; Gonzalez et al., 2018; Montero, Diaz-Montero, Kyriakopoulos, Bronte, & Mandruzzato, 2012). Three key events regulate MDSC activity during cancer progression: deregulated myelopoiesis, migration of MDSCs to tumor, and MDSC activation. Impaired myelopoiesis amplifies the production of MDSCs in the BM that are consequently recruited to the tumor site by tumor-derived and stromal-cell-derived factors (Condamine & Gabrilovich, 2011; Y. Wang, Ding, Guo, & Wang, 2019). The prevailing factors in the recruitment of PMN-MDSCs and M-MDSCs to the tumor are similar to those regulating the recruitment of granulocytes and monocytes. M-MDSCs and monocytes are mainly recruited by chemokines produced by tumor cells, including CCL2, CCL5, and CSF1. The migration of PMN-MDSCs is regulated by the secretion of C-X-C chemokines including CXCL1, CXCL5, CXCL6, CXCL8, and CXCL12 by tumor cells (Lim, Kim, & Poh, 2020) (Fig. 1). For example, gastric, ovarian, and breast cancer cells secrete CCL2 resulting in the recruitment of MDSCs (B. Huang et al., 2007). In addition, CCR2+ MDSCs are reported to increase tumor growth in mice bearing colorectal tumors (Katoh et al., 2013). CXCL1 is another chemokine expressed in colorectal cancer, and it exerts chemoattractant activity on CXCR2+ MDSCs (Katoh et al., 2013). MDSCs also express chemokine receptor CCR5 that has been reported to contribute to the migration of MDSCs to the tumors (Blattner et al., 2018).

The differentiation and development of MDSCs recruited in the TME can be skewed in different directions based on the environmental factors (Tannenbaum et al., 2019). Hypoxia in the TME is also a significant factor stimulating the recruitment of MDSCs and establishing an angiogenic and immunosuppressive microenvironment to promote tumor metastasis (Corzo et al., 2010; Park et al., 2019). Several studies have shown that hypoxia-inducible factor 1 (HIF 1α) plays a vital role in generating M2 TAMs from Ly6C^hi^ monocytes inside a tumor (Laoui et al., 2014; Van Overmeire, Laoui, Keirsse, & Van Ginderachter, 2014). Different cytokines and immunomodulatory proteins are also reported to differentiate MDSCs into M2-type TAMs in hypoxic conditions (Corzo et al., 2010). TAMs can also be derived from Ly6C^hi^ or Ly6C^hi^CX3CR1^lo^ or Ly6C^+^CCR2^+^ monocytes or tissue-resident macrophages (Cortez-Retamozo et al., 2012; Movahedi et al., 2010; Movahedi & Van Ginderachter, 2016; Y. Zhu et al., 2017). Emerging studies have shown that M-MDSCs can differentiate into M2-TAMs in the TME to promote tumor growth (Corzo et al., 2010; Qian et al., 2011; Zhou et al., 2015). Kwak et al have recently reported that macrophages differentiated from M-MDSCs are highly immunosuppressive and constitutively express S100A/9 protein, while tissue-resident macrophages and macrophages differentiated from monocytes lack the expression of this protein (Kwak et al., 2020). The differentiation of G-MDSCs in the TME is not extensively studied as the lifespan of this subset is shorter than the M-MDSC subset (Condamine et al., 2014). Overall, the differentiation of MDSCs in the TME is complex and further studies are needed to better understand factors driving differentiation of M-MDSCs and PMN-MDSCs in the TME.

MDSCs can be activated in the TME by multiple different mechanisms. In particular, the signal transducer and activation of transcription (STAT) family of transcription factors, namely STAT 3 promotes the activation and expansion of MDSCs (H. J. Ko & Kim, 2016). STAT3, together with GMCSF, GCSF, and VEGF, increases MDSC levels within the tumor and inhibit the differentiation of MDSCs into neutrophils and monocytes (Nefedova et al., 2004; Trikha & Carson 3rd., 2014). A study by Kumar et al has shown that decreased expression of STAT3 can promote the differentiation of M-MDSCs into TAMs with M2 or immunosuppressive phenotype (Kumar et al., 2016). STAT3 also up-regulates S100A8/9 pro-inflammatory proteins, which regulate the accumulation of MDSCs in the TME (Sinha et al., 2008). S100A8/9 proteins are also reported to block the differentiation of myeloid progenitor cells in breast and gastric cancer (Arai et al., 2008; L. Wang et al., 2013).

## 4. Role of MDSCs in angiogenesis and immunosuppression

**Fig. 2.**
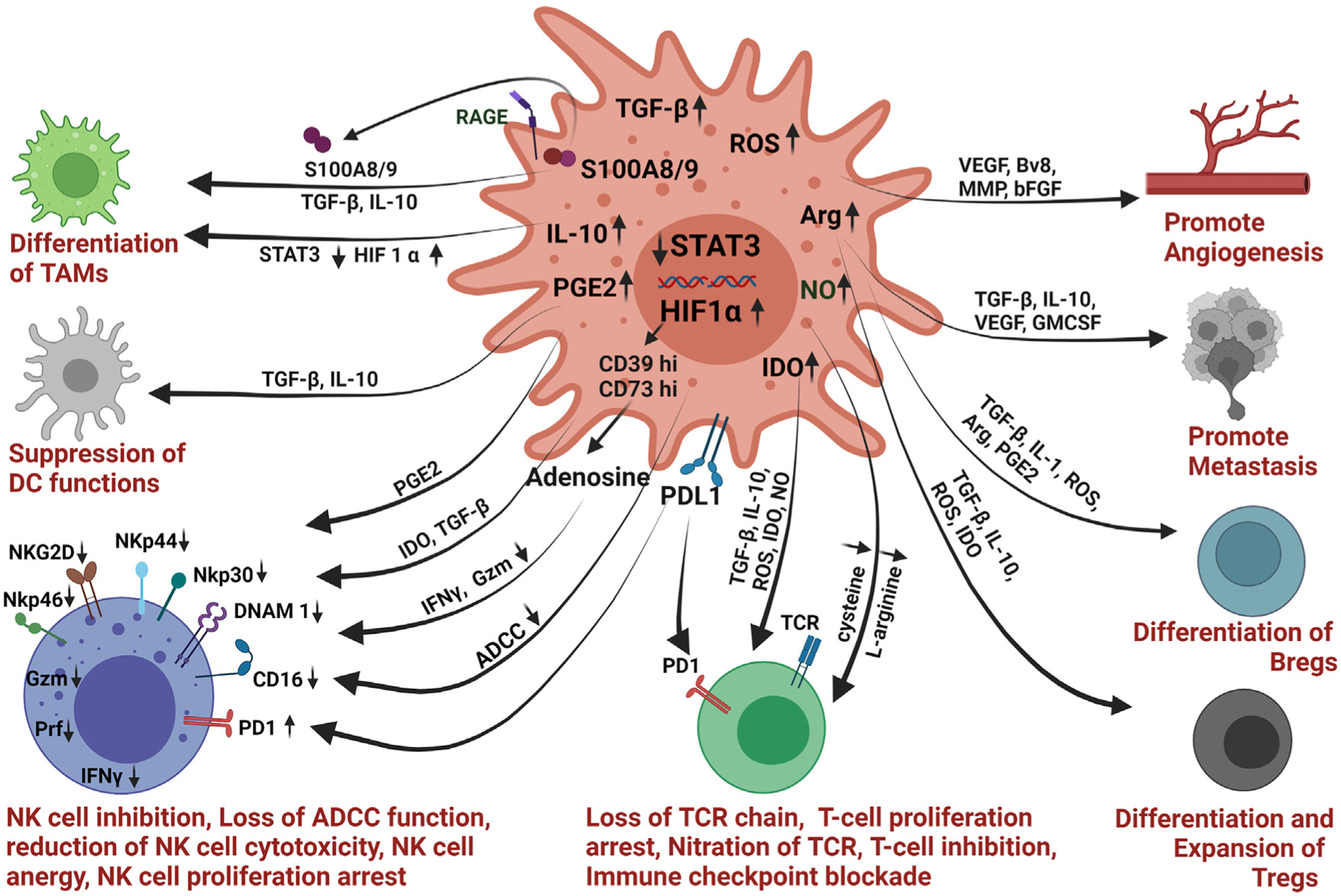
Schematic of various strategies employed by MDSCs to promote tumor growth and immunosuppression. MDSCs use various mechanisms to induce angiogenesis, metastasis, to promote differentiation of M2-type macrophages, T_reg_ cells, B_reg_ cells and to suppress the functions of T cells, NK cells, and DC. MDSCs impede the functions of NK cells by secreting TGFβ, IDO, ROS, NO, and PGE2. The fig. is created with Biorender.com.

Activated MDSCs employ various mechanisms to promote angiogenesis, immunosuppression, and tumor growth (Fig. 2) (Groth et al., 2019; Law et al., 2020; Tesi, 2019). In the TME, MDSCs trigger and sustain tumor angiogenesis by secreting several factors like basic FGF (bFGF), VEGF, matrix metalloproteinase (MMPs), and prokineticin (Bv8) (Ahn et al., 2014; Baniyash, 2016; Fleming et al., 2018). MDSCs secrete TGFβ, IL-10, VEGF, and GMCSF to support tumor metastasis (Y. Wang et al., 2019). MDSCs also mediate immunosuppression by inhibiting CD4+, CD8+ T cells, and NK cells, resulting in immune escape and progression of cancer (Gabrilovich & Nagaraj, 2009; Joshi & Durden, 2019). The various mechanisms used by MDSCs to promote immunosuppression include: 1) reduction of amino acids needed for T cell proliferation and activation (Srivastava, Sinha, Clements, Rodriguez, & Ostrand-Rosenberg, 2010); 2) release of immunosuppressive cytokines like interleukin-10 (IL10) and TGFβ to promote differentiation of regulatory B (B_reg_) cells and T_reg_ cells (B. Huang et al., 2006; C. R. Lee et al., 2016; Shen et al., 2018); 3) recruitment of regulatory T cells (T_regs_) (Pan et al., 2010); 4) engaging with inhibitory receptor PD1 to block T cell/NK cell activity (Y. Jiang, Chen, Nie, & Yuan, 2019; Pesce et al., 2019), 5) down regulation of NK cell activating receptors (Della Chiesa et al., 2006) 6) down-regulation of STAT-3 and increase in HIF1α to induce differentiation of M2 macrophages (Eruslanov, Daurkin, Ortiz, Vieweg, & Kusmartsev, 2010); 7) secretion of S100A8/9, which promotes M2 macrophage polarization and chemotaxis of MDSCs in the TME (Cheng et al., 2019), 8) suppression of antigen presenting functions of dendritic cell (DC) (Ugolini et al., 2020) (Fig. 2). MDSCs are also reported to secrete exosomes packed with tumor-promoting factors like TGFβ, IL-10, MMP, and micro-RNAs that are transported to tumor sites and induce immunosuppression (Xiang et al., 2009).

The role of MDSCs in inhibiting T cell responses has been studied extensively and reviewed in detail elsewhere (Groth et al., 2019; Law et al., 2020; Tesi, 2019). In brief, the accumulation and expansion of MDSCs in TME consume amino acid cysteine, resulting in the suppression of T cell proliferation (Ostrand-Rosenberg, 2010). T cell function is highly dependent on the exogenous source of cysteine as it cannot be synthesized *denovo* by T cells (Ostrand-Rosenberg, 2010). MDSCs can further reduce essential amino acids from the TME by catabolizing tryptophan and L-arginine (Fletcher et al., 2015). MDSCs express enhanced levels of Arg and iNos to inhibit the function of T cells by depleting L-arginine in the TME (Bingisser, Tilbrook, Holt, & Kees, 1998; Bronte & Zanovello, 2005). L-arginine depletion and NO generation can inhibit major histocompatibility complex II (MHCII) expression, reduce the expression of the T-cell receptor (TCR) chain resulting in inhibition of T cell proliferation and function (Harari & Liao, 2004; Nagaraj et al., 2007; Rivoltini et al., 2002; Rodriguez, Quiceno, & Ochoa, 2007). In addition, an increase in reactive oxygen species (ROS) can abrogate antigen-specific responses in CD8+ T cells (Kusmartsev & Gabrilovich, 2003). High levels of ROS can also reduce the expression of the TCR chain resulting in T cell deactivation (Kusmartsev, Nefedova, Yoder, & Gabrilovich, 2004). Moreover, secretion of interleukin 10 (IL-10) and TGF-β by MDSCs induce expansion of T_regs_ (Hart, Byrne, Molloy, Usherwood, & Berwin, 2011; Marvel & Gabrilovich, 2015). MDSCs also express indoleamine 2, 3 dioxygenase (IDO) to convert L-tryptophan into keyneurine, leading to inhibition of T cell proliferation. In addition, MDSCs express immunostimulatory receptor CD40 to block T cell proliferation (Pan et al., 2010). MDSCs also express PDL1 to inhibit anti-tumor T cell responses. The up-regulation of PDL1 expression in MDSCs is associated with the increased hypoxia-inducible factor (HIF1α) (Noman et al., 2014). Based on the sub-type, MDSCs use different mechanisms to abrogate anti-tumor immunity (Law et al., 2020; Nagaraj et al., 2007). M-MDSCs mainly employ non-specific inactivation of T-cells by expressing higher levels of Tgfβ, Arginase (Arg1), and iNos. While PMN-MDSCs produce high levels of ROS and mediate immunosuppression by direct cell-cell contact with T cells, reducing antigen-specific T cell responses without affecting responses to non-specific stimuli. The various strategies employed by MDSCs to inhibit the function of NK cells are discussed later in this review.

## 5. NK cells: Development and subtypes

Natural killer cells are cytokine-producing effector innate lymphoid cells that lyse and kill target cells to eradicate tumors (Herberman, Nunn, & Lavrin, 1975). NK cells are developed in bone marrow from CD34+ hematopoietic stem cells (Cichocki, Grzywacz, & Miller, 2019). The maturation of NK cells is mediated by several cytokines, among which interleukin-15 (IL-15) is an important factor promoting the differentiation of NK cell lineage from common lymphoid progenitor cells (Abel, Yang, Thakar, & Malarkannan, 2018; Crinier, Narni-Mancinelli, Ugolini, & Vivier, 2020). NK cells are commonly present in PB, BM, and spleen but can be found in the lungs, skin, liver, and lymph nodes (Bjorkstrom, Ljunggren, & Michaelsson, 2016; Shi, Ljunggren, La Cava, & Van Kaer, 2011).

These innate cells can be identified by the lack of TCR and related CD3 molecules but recognized by the expression of neural cell adhesion molecule (NCAM 1 or CD56) and the cluster of differentiation molecule CD16 (aka FcγRIIIA) (Cooper, Fehniger, & Caligiuri, 2001; Freud, Mundy-Bosse, Yu, & Caligiuri, 2017). The surface expression of CD56 antigen varies in NK cells and based on expression, NK cells can be classified into two subtypes: CD56^bright^ and CD56^dim^. CD56^dim^ NK cells are defined as “mature” NK cells and constitute 90% population of peripheral blood NK cells, while CD56^bright^ cells are regarded as “immature” NK cells and usually predominate in tissues and secondary lymphoid organs. These two subtypes show differences in the secretion of cytokines and the efficiency of killing target cells (Crinier et al., 2020). CD56^dim^ cells have higher cytotoxic potential, while CD56^bright^ cells are poorly cytolytic but can induce secretion of IFNγ and tumor necrosis factor (TNFα). It is believed that immune responses mediated through NK cells were maximal at the first attack, and these responses are not augmented on subsequent exposure of the same target. Recent findings suggest that NK cells acquire long-term memory (Nikzad et al., 2019; Paust, Blish, & Reeves, 2017). However, further studies are needed to demonstrate this phenomenon conclusively.

## 6. NK cells activation and their anti-tumor functions

NK cells are cytokine-producing innate immune cells with anti-tumor functions (Chiossone, Dumas, Vienne, & Vivier, 2018; Vacca et al., 2019). As reviewed before, NK cells express various activating and inhibitory receptors (Pegram, Andrews, Smyth, Darcy, & Kershaw, 2011). The activating receptors present in NK cells include natural killer group 2, member D (NKG2D), DNAX accessory molecule 1 (DNAM-1), and natural cytotoxic receptor (NCR) (i.e., NKp30, NKp44, and NKp46). In humans, NKG2D identifies major histocompatibility complex (MHC) class I chain-related protein A (MICA) and B (MICB) and UL-16 binding proteins (S. Bauer et al., 1999; Wensveen, Jelencic, & Polic, 2018). Costimulatory adhesion receptor DNAM 1 binds PVR and nectin 2, this binding promotes tumor identification by NK cells (Lakshmikanth et al., 2009). NCRs are group of activating receptors that recognize ligands present in viruses, bacteria, and tumor cells. Human NK cells express NKp30, NKp44, and NKp46, while mouse NK cells only express NKp46. NK cells also express CD16 (FCγRIIIA) receptor that recognizes Fc regions of antibody molecules coated opsonized target cells. The inhibitory receptors regulating NK cell activation are classified into two types: HLA-specific and non-HLA-specific inhibitory receptors. The HLA-specific inhibitory receptors include killer Ig-like receptors (KIR)/CD158, CD94/NKG2A/NKG2C, LAG-3, and LIR1. The non-HLA-specific receptor includes SIGLEC-7, TIM-3, TIGIT, PD-1, and IRP-60 (Sivori et al., 2020).

NK cells identify the expression of MHC I on murine cells and human leukocyte antigen class I (HLA 1) molecules on human cells. This interaction prevents NK cells from killing normal cells. In contrast to transformed cells, normal cells express high levels of MHC I and usually escape NK cell immune attack. NK cells recognize cells with low MHC I expression and increased expression of activating ligands such as NKG2D ligand, Nkp30, and NKp44. The recognition of these receptors induces activation of NK cells resulting in the secretion of cytokines and tumor cell lysis (E. O. Long, Kim, Liu, Peterson, & Rajagopalan, 2013; Topham & Hewitt, 2009). Activated NK cells kill target cells by releasing cytotoxic granules containing perforin or granzyme (Gzm) (Paul & Lal, 2017; Sanseviero, 2019). Perforin produces pores on the cancer cells and granzyme enters into the cells to destroy it (Pardo, Balkow, Anel, & Simon, 2002). NK cells are also known to express TNF receptor ligands TRAIL, FASL, and TNFR. Interaction of these receptors with their ligands on the target cells induces apoptosis of target cells (Screpanti, Wallin, Ljunggren, & Grandien, 2001; Sonar & Lal, 2015; Thorburn, 2004).

The infiltration of NK cells in solid tumors is very poor, and the TME impacts the permeability of these cells in the tumors (Guillerey, Huntington, & Smyth, 2016; Melaiu, Lucarini, Cifaldi, & Fruci, 2019; Rossi, Trindade, & Souza-Fonseca-Guimaraes, 2020; Russick, Torset, Hemery, & Cremer, 2020). Recent reports suggest the poor infiltration of NK cells in colorectal carcinoma and melanoma (Halama et al., 2011; Tartter, Steinberg, Barron, & Martinelli, 1987). On the contrary, some tumors show higher infiltration of NK cells in the tumors, and their increased number in tumor tissue is linked with improved survival. In head and neck cancers and renal carcinomas, the presence of NK cells in tumors is associated with better survival (Eckl et al., 2012; Schantz & Ordonez, 1991). In breast cancers, tumor-infiltrating NK cells are considered predictors of response to anti-human epidermal growth receptor 2 (anti-HER2) mAb therapy (Salgado et al., 2015). However, in non-small lung cancer (NSCLC), infiltration of NK cells has no impact on clinical outcomes (Carrega et al., 2008).

## 7. Interactions between MDSCs and NK cells

Several studies in mouse models and cancer patients suggest an inverse correlation between NK cells and MDSCs (Greene et al., 2020; Parihar et al., 2019; Sato et al., 2015; Shou et al., 2016). Nevertheless, the involvement of MDSCs in controlling NK cell functions remains an underexplored topic. NK cells communicate with tumor cells and other stromal cells using three main mechanisms: 1) cell-cell contact; 2) secretion of soluble factors, and 3) release of extracellular vesicles (Di Pace et al., 2020; H. Li, Han, Guo, Zhang, & Cao, 2009; Z. Li et al., 2012). MDSCs employ multiple methods to modify NK cell frequency and activity (Fig. 2) (Bruno et al., 2019; Tumino et al., 2021; Zalfa & Paust, 2021). Several studies have shown the accumulation of CD11b^+^Gr1^+^ MDSCs in the spleen of tumor-bearing mice resulting in reduced NK cell cytotoxicity by either down-regulating the expression of activating receptors NKG2D, NKp30 or by reducing the production of IFN, perforin (Bruno et al., 2019; Greene et al., 2020; H. Li et al., 2009).

Preclinical studies in mice suggest that cell-cell contact is required for MDSC-mediated inhibition of NK cells, and this effect is mainly facilitated by TGFβ (Z. Li et al., 2012). Membrane-bound TGFβ reduces expression of NKG2D and IFN gamma production in NK cells leading to impaired NK cell cytotoxicity in a murine liver cancer model (H. Li et al., 2009). The impaired function of NK cells in mice bearing orthotopic liver tumors could be restored by inhibiting TGFβ on MDSCs. This study demonstrates that cell to cell contact between MDSC and NK cells is essential to induce NK cell anergy (H. Li et al., 2009). A recent study by Greene et al have shown that CXCR2+ MDSCs positively accumulate in the spleens of mice bearing head and neck tumors and suppress NK cell function through TGFβ and production of H_2_O_2_ (Greene et al., 2020). IL-33 is also reported to mediate MDSC-NK cell interaction. IL-33 is mainly secreted by endothelial and epithelial cells under stress conditions, and depending on the tumor microenvironment, it promotes or blocks tumor growth in murine models (Fournie & Poupot, 2018; J. X. Shen, Liu, & Zhang, 2018). IL-33 can recruit MDSCs and TAMs to support tumor growth but can also stimulate the infiltration of cytotoxic NK cells and CD8+T cells to regress tumor growth (Fournie & Poupot, 2018). Based on its pro-tumor and anti-tumor functions, in humans, IL33 is correlated with poor prognosis in glioma and hepatocellular carcinoma (W. Wang, Wu, Ji, & Wu, 2020; J. Zhang, Wang, Ji, Ding, & Lu, 2017) while it is associated with better prognosis in lung cancer and colorectal cancer (O’Donnell et al., 2016; Yang et al., 2018).

Recently, a novel subset of MDSCs which lack Ly6C expression has been reported (Elkabets et al., 2010). This subset of MDSCs expands in the TME in the presence of IL-1β and demonstrates strong inhibitory potential against NK cells by downregulating the NKG2D receptor. MDSCs can also downregulate the expression of CD247 on the NK cell surface, which promotes NK cell anergy. CD247 is a crucial subunit of NCRs; NKp30, NKp46, and CD16. In patients with HCC, MDSCs interact with the NKp30 receptor to inhibit the functions of NK cells (Hoechst et al., 2009). The study shows that MDSC cocultured with NK cells inhibits the cytotoxicity of NK cells in patients with HCC. This suppression of NK cells by MDSCs is not mediated through arginase or inducible nitric oxide synthase or IDO. However, it is mediated through the NKp-30 receptor specifically in a cell contact-dependent manner.

A study by Naush et al demonstrated that co-culture of MDSCs with NK cells impairs the cytotoxicity of NK cells, and this effect is mediated mainly by decreased expression of NKG2D ligand (Nausch, Galani, Schlecker, & Cerwenka, 2008). MDSCs are reported to reduce the anti-tumor activities of NK cells, and long-lasting inflammation is known to enhance these effects. Several proinflammatory cytokines present in the TME like IFNγ increase the expansion of MDSCs which in turn secrete IL-10 (Guo et al., 2012). While IL-10 is known to inhibit the functions of T cells, it can also inhibit NK cells and promote T_H_2 type immune responses (Yaseen, Abuharfeil, Darmani, & Daoud, 2020). Another inflammatory mediator that may be associated with MDSC mediated regulation of NK cells is Prostaglandin E2 (PGE2). PGE2 is produced by the COX2 signaling cascades and is associated with tumor growth, angiogenesis, and induction of immunosuppression (Harris, Padilla, Koumas, Ray, & Phipps, 2002). PGE2 plays an important role in generating MDSCs from monocytes and inhibiting NK cell activation in TGFβ dependent manner (Mao et al., 2014). Mao et al. observed a similar phenomenon when NK cells were co-cultured with M-MDSCs isolated from melanoma patients (Mao et al., 2014).

MDSCs can also secrete various soluble factors like nitric oxide (NO) inducible nitrogen-oxygen synthase (iNOS), per oxynitrate, reactive oxygen species (ROS), and ARG1 to promote immunosuppression and to inhibit the activation of NK cells (Gabrilovich, 2017). NO is a gaseous transmitter that plays a key role in inflammation and cancer. The autocrine generation of NO by NK cells positively influences the function of NK cells while MDSC-derived NO significantly impacts the cytotoxic functions of NK cells (Ying & Hofseth, 2007). Stiff et al have shown that NO generated by MDSCs impairs Fc mediated function and downstream effector functions of NK cells, including antibody-dependent cellular cytotoxicity (ADCC) and secretion of IFNγ and TNFα (Stiff et al., 2018). MDSC-mediated generation of ROS and ARG 1 also impairs the function of NK cells in cancer models *in vivo* (Groth et al., 2019). Moreover, MDSCs can secrete adenosine in TME by inducing the expression of CD39 in tumors (Ryzhov et al., 2014). Adenosine is an immunosuppressive molecule that inhibits NK cell cytotoxicity by limiting IFNγ/TNFα release (Raskovalova, Lokshin, Huang, Jackson, & Gorelik, 2006). Adenosine signaling also plays a crucial role in determining the maturation of NK cells (Cekic, Day, Sag, & Linden, 2014).

The STAT family of transcription factors modulates functions of innate and adaptive immune cells (H. Yu, Pardoll, & Jove, 2009). STAT1 and STAT5 is involved in anti-tumor responses, while STAT3 and STAT6 are known to mediate immunosuppression in the TME (H. Yu et al., 2009). STAT3 activation in MDSCs has been demonstrated to induce activation of NF-KB, resulting in the release of IDO (Sui et al., 2014; X. Sun, Sui, Zhang, Tian, & Zhang, 2013; J. Yu et al., 2014). IDO is a heme-containing enzyme that regulates the tryptophan catabolism into kynurenine (Lob, Konigsrainer, Rammensee, Opelz, & Terness, 2009; Munn & Mellor, 2016). Kynurenine production inhibits the proliferation of T cells and NK cells (Godin-Ethier et al., 2009). MDSCs are considered as main producers of IDO in the TME (J. Yu et al., 2013). IDO produced by MDSCs reduces the expression of NKG2D, DNAM1, and NCR and blocks the activation of NK cells (J. Zhang et al., 2018). Various studies have shown that STAT3 inhibition can increase tumor recognition by NK cells (Sui et al., 2014; X. Sun et al., 2013). A study by Sui et al have shown that STAT-3 targeted tumor-bearing mice showed tumor regression due to NK cell activity (Sui et al., 2014). The authors have shown that NK cells isolated from treated tumors have up-regulated expression of NK activation markers CD69, NKG2D, IFNγ, perforin, and granzyme B.

Checkpoint inhibitors targeting the PD1/PDL1 axis have shown promising results in treating various cancers (Joshi & Durden, 2019; Sharma & Allison, 2015). PD1 is expressed mainly by T cells but activated NK cells also express PD1 receptor (Barry et al., 2018; Hsu et al., 2018; Mariotti et al., 2019; Quatrini et al., 2020). The expression of PDL1, a ligand of PD1, is high on tumor-infiltrating MDSCs, and hence blocking PD1-PDL1 interactions can restore the activity of T cells and NK cells. Taken together, many molecules and pathways involved in MDSC mediated inhibition of T-cells may also impact and inhibit NK cells as well.

## 8. NK-Cell-based immunotherapy approaches

Several landmark reviews are available that highlight the current approaches used in NK-cell-based immunotherapy (Fang, Xiao, & Tian, 2017; Hu, Wang, Huang, Sui, & Xu, 2019; Myers & Miller, 2021). Here we focus on three approaches: cytokine-based approach to expand NK cells; adoptive transfer of unmanipulated/unmodified NK cells, and adoptive transfer of genetically manipulated/modified NK cells.

### 8.1. Cytokine-induced human NK cell expansion and activation

Several preclinical and clinical studies have shown that cytokines can promote the differentiation, proliferation, and activation of NK cells (Hu et al., 2019; Konjevic, Vuletic, Mirjacic Martinovic, Larsen, & Jurisic, 2019). Cytokine-mediated NK cell activation is frequently used and is currently under investigation (Hu et al., 2019; Myers & Miller, 2021). Various Cytokines like IL-2 (Dhupkar & Gordon, 2017), IL-15 (Daud et al., 2008; Perez-Martinez et al., 2015), IL-12 (Little et al., 2006), and IL-18 (Robertson et al., 2008; S. Srivastava et al., 2013) either alone or together with other agents, have been used to improve NK cell functions (Floros & Tarhini, 2015). In numerous studies, IL-2 or IL-15 is used as a supplement for the expansion of NK cells ex *vivo* (Miller et al., 2005). IL-2 is the most common cytokine currently used to enhance NK cell cytotoxicity in preclinical and clinical studies (S. Srivastava et al., 2013). A Phase 3 clinical trial combining anti-GD2 mAb with IL-2, GMCSF, and isotretinoin has shown promising results in improving event-free survival in patients with relapsed and refractory neuroblastoma (A. L. Yu et al., 2010). However, high dose IL-2 can cause adverse effects due to the expression of IL2 affinity receptor IL-2Rαβγ (Ito et al., 2014). These obstacles lead to the generation of a new variant of IL-2 known as “IL-2 superkine” (super-2). This engineered “superkine” has increased affinity for IL2/15R subunit present on NK cells (Levin et al., 2012). IL-15 is another cytokine used in NK cell immunotherapy and is considered as a superior substitute to IL2 as it specifically activates immature and mature NK cells (Waldmann, 2006). There are several ongoing clinical trials exploring rhIL-15 together with antibodies (Conlon et al., 2015; Miller et al., 2018). ALT-803 is a superagonist of IL-15 with strong potential to activate NK cells (Wong, Jeng, & Rhode, 2013). ALT-803 is currently being evaluated in several clinical trials together with T cell and NK cell therapy (Romee et al., 2018; Wrangle et al., 2018).

### 8.2. Adoptive transfer of unmanipulated/unmodified NK cells

NK cells can kill target cells using multiple mechanisms; hence the adoptive transfer of NK cells hasthe been investigated in various preclinical and clinical studies (Fang et al., 2017; Myers & Miller, 2021). For adoptive transfer, NK cells can be acquired from different sources. These sources include autologous NK cells (cells are obtained from same donor), allogeneic NK cells (cells obtained from the different donor), NK cell lines, induced pluripotent stem cell (iPSC)-derived NK cells and umbilical cord blood (UCB)-derived NK cells. Adoptive transfer of autologous NK cells has been tested in several clinical trials to treat patients with colon cancer, lymphoma, lung cancer, and breast cancer (Geller et al., 2011; Krause et al., 2004; Parkhurst, Riley, Dudley, & Rosenberg, 2011). However, a very limited anti-tumor effect was observed due to the limited activation of NK cells (Ruggeri et al., 2002). In addition, the NK cells derived from cancer patients were already immunosuppressed and displayed impaired function, with limited antitumor effector activity. Several studies have shown that PBMC derived *ex vivo* activated alloreactive NK cells can overcome these issues. As a result of this, allogeneic NK cells are investigated in various clinical trials of solid tumors as well as hematological malignancies (Miller et al., 2005).

A large number of NK cells for immunotherapy can also be produced by using NK cell clonal cell lines such as NK-92, NK-YS, NKG, YTS, YT, NKL, all of which can quickly grow in cell culture. Among these cell lines, NK-92 is the only cell line used in clinical trials. The safety of NK-92 cells has been evaluated in various clinical trials (Arai et al., 2008; Boyiadzis et al., 2017). However, the clinical efficacy of NK-92 cells is minimal, and it is mainly due to their less stability *in vivo* (Suck et al., 2016). To overcome this obstacle, a variant NK-92 cell line known as high-affinity natural killer cells (haNK), has been developed. (Jochems et al., 2016). This variant cell line can do antibody-dependent cellular cytotoxicity (ADCC) and circumvent the need to supplement IL-2 in culture. NK cells can also be obtained from human pluripotent stem cells (hPSCs), which includes induced pluripotent stem cells (iPSCs), and human embryonic stem cells (hESC), (Eguizabal et al., 2014; Knorr et al., 2013). NK cells generated from stem cells have a strong proliferative capability and closely resemble primary NK cells in terms of proliferation which makes them a potential candidate for clinical use (Knorr et al., 2013; Woll et al., 2009). Umbilical cord blood-derived (UCB) NK cells have recently gained more attention for their use in the adoptive transfer of NK cells. Recently it is shown that cryopreservation had no effects on the effector functions of UCB-derived NK cells and hence these cells can also be used efficiently for NK cell therapies (Nham et al., 2018; Woll et al., 2009; Woll, Martin, Miller, & Kaufman, 2005).

### 8.3. CAR-NK cells for cancer immunotherapy

Chimeric antigen receptors (CAR)-T cell therapy has shown promising results in the treatment of patients with blood cancer, but in solid tumors, the efficacy of CAR-T is restricted due to highly immunosuppressive TME that hampers the activity of immune cells (Chow et al., 2019; June & Sadelain, 2018). The high expression of PD1 and PDL1 by T cells present in the TME also suppresses the activity of CAR-T cells in solid tumors (Newick, O’Brien, Moon, & Albelda, 2017). CAR-T therapies are very costly and labor-intensive, and their use is often restricted due to graft vs. host disease (GvHD) (Ghosh et al., 2017). In addition, cytokine release syndrome and other adverse effects associated with CAR-T therapy limited its clinical applications. Genetic modification of NK cells with CARs has garnered a lot of attention recently. NK cells are superior alternative to T cells for eliminating solid tumors as 1) they express low levels of PD1 (Barry et al., 2018), 2) can be easily isolated and generated in large numbers as “off-shelf” products (Siegler, Zhu, Wang, & Yang, 2018), 3) cytokines produced by NK cells is IFNγ and GMCSF and they don’t promote cytokine release syndrome.

The basic structural design of CAR-NK is similar to CAR-T. It comprises chimeric antigen receptors, transmembrane hinge region and a co-stimulatory signaling domain (Oberschmidt, Kloess, & Koehl, 2017; Zhang et al., 2017). Four generations of CARs are reported. The first generation of CARs contain the CD3ζ domain, second and third-generation CARs contain costimulatory molecules like 4-1BB, CD28, and CD134, and fourth-generation CARs are designed to secrete IL12 (Rafei, Daher, & Rezvani, 2021).

Recently, NKG2D.ζ, NK cell-bearing CAR is generated which targets MDSCs in neuroblastoma tumors (Parihar et al., 2019). Several CAR-NK cells directed against different antigens have been developed and have been evaluated in preclinical and clinical studies for solid tumors (Fang et al., 2017; Myers & Miller, 2021). Several CAR-NK clinical trials are ongoing as previously reviewed (Zalfa & Paust, 2021). Despite the scientific advantages, there are still challenges that need to be diligently addressed to improve the success of NK-cell immunotherapies. MDSC-mediated immunosuppression reduces the efficacy of NK-cell-based therapies in solid tumors and hence in the next section, we discuss how MDSC targeting strategies can be strategically combined with NK cell-based immunotherapies to enhance the cytotoxic potential of NK cells.

## 9. MDSC targeting strategies to enhance NK cell immunotherapies

**Fig. 3.**
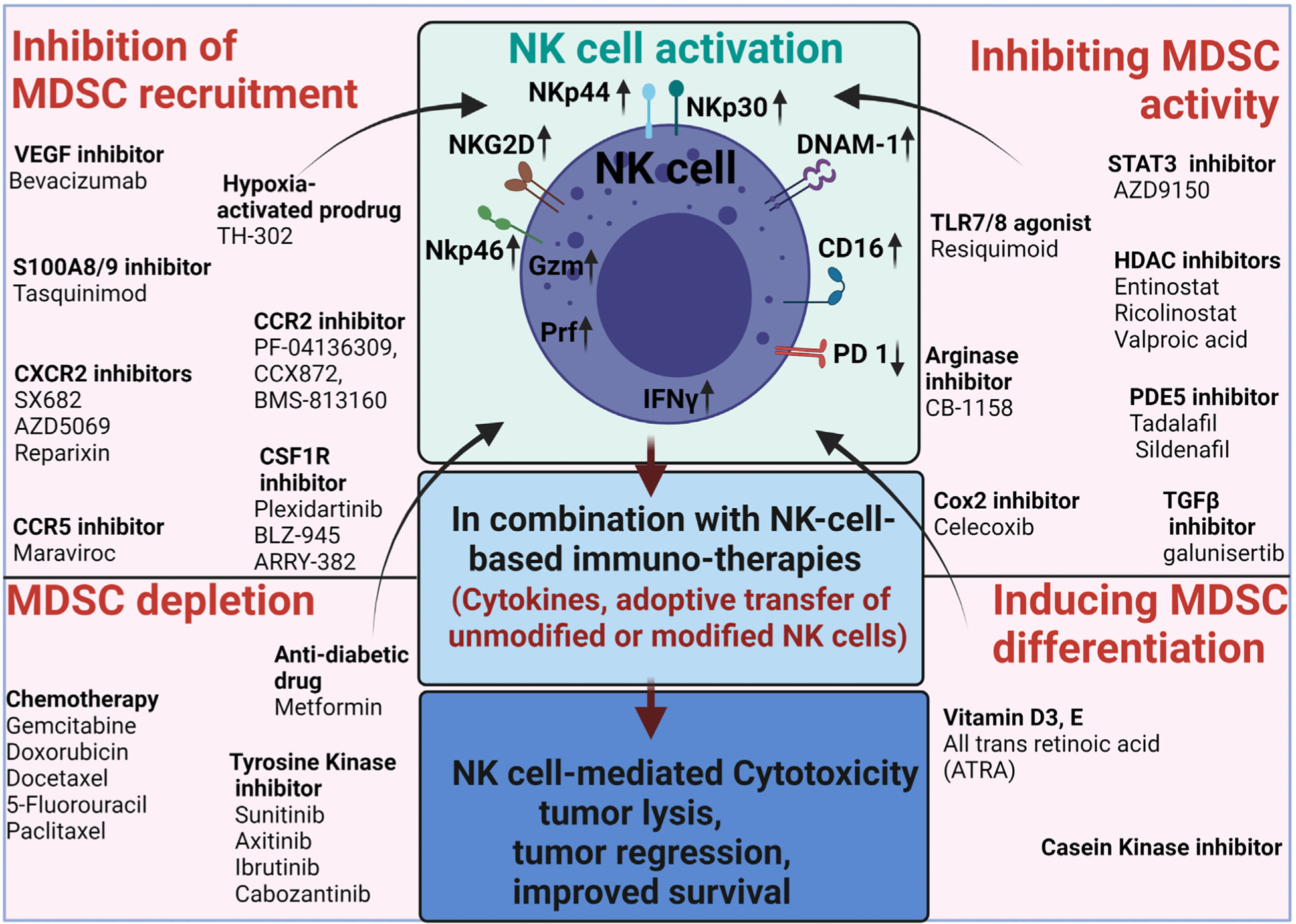
Strategies to enhance NK cell immunotherapy by targeting MDSCs. MDSCs are potent immunosuppressive cells in the TME. Hence targeting these innate immune cells can enhance NK-cell targeted immunotherapy. MDSCs can be targeted by blocking their recruitment in the TME or inducing differentiation of MDSC, or depleting MDSCs or blocking immunosuppressive functions of MDSCs. Although NK cells have provided clinical benefit in some types of cancer, it is essential to combine MDSC targeted therapies with NK cell therapies to increase NK cell activation, cytotoxicity, and survival. The fig. is created with Biorender.com.

Several MDSC targeting strategies have been tested in preclinical and clinical studies to inhibit immunosuppression and enhance the cytotoxicity of NK cells. Specifically, four strategies have been tested 1) targeting recruitment and trafficking of MDSCs; 2) depletion of MDSCs 3) inducing MDSC differentiation; and 4) inhibition of MDSC immunosuppressive functions (Fig. 3). Clinical trials ongoing with MDSC targeting agents either alone or in combination with other agents have been recently reviewed (Fleming et al., 2018) Here, we discuss various strategies currently used to reduce MDSC-mediated immunosuppression and how these strategies can be combined with the latest NK cell therapies to improve patient survival. The preclinical studies and clinical trials with MDSC targeted agents either alone or in combination with NK cell immunotherapies are listed in Table 1.

### 9.1. Targeting recruitment and trafficking of MDSCs

MDSCs mainly perform their immunosuppressive functions within the TME; hence various studies have been conducted to block the recruitment and migration of MDSCs to the tumors. Tumor cells are the primary source of VEGF in the TME. VEGF stimulates angiogenesis and acts as a chemoattractant for MDSCs. A study by Koinis et al. has shown that VEGF attracts MDSCs from the BM to the tumor bed and is involved in the expansion of MDSCs in the NSCLC model (Koinis et al., 2016). Bevacizumab, an anti-VEGF recombinant human mAb has shown significant efficacy in reducing intra-tumoral MDSCs in preclinical and clinical studies (Feng et al., 2018; Ferrero et al., 2016; Isambert et al., 2018). For example, bevacizumab treatment reduced the frequency of MDSCs in the peripheral blood of patients with NSCLC (Koinis et al., 2016). In another study, bevacizumab combined with EGFR tyrosine kinase inhibitor reduced circulating S100A9 positive MDSCs and improved progression-free survival in patients with EGFR mutant lung adenocarcinoma (Feng et al., 2018). In Phase 1 clinical trial (NCT02669173), bevacizumab treatment combined with capecitabine decreased the frequency of MDSCs in glioblastoma patients (Peereboom et al., 2019). Similarly, in patients with colorectal cancer, bevacizumab combined with 5-fluorouracil and oxaliplatin showed a decrease in circulating MDSCs (Limagne et al., 2016). Bevacizumab has been tested with NK cell therapies in preclinical and clinical studies. Bevacizumab combined with umbilical-cord-derived NK cells, enhanced the extravasation of adoptively transferred NK cells in mice xenografted with human LoVo colorectal tumors (Xu et al., 2019). In Phase I and 2 clinical trials, bevacizumab was combined with allogenic NK immunotherapy (NCT02857920) for metastatic solid tumors, and no outcome was reported for this trial. These two reports do not provide evidence if bevacizumab reduces the infiltration of MDSCs or modulates the function of MDSCs to enhance the activation of NK cells. However, these studies suggest that bevacizumab treatment can enhance the cytotoxicity of NK cells. Hence, studies using a combination of bevacizumab with adoptive transfer of unmodified/modified NK cells can be explored in the near future.

HIF 1α is released in the TME and plays an important role in the accumulation of MDSCs and differentiation of MDSCs into TAMs (Corzo et al., 2010; Joshi et al., 2020). In the hepatocellular carcinoma model, hypoxia promotes stabilization of HIF1α which induces increased expression of ectoenzyme, ectonucleoside triphosphate diphosphohydrolase 2 (ENTPD2/CD39L1) in cancer cells (Chiu et al., 2017). ENTPD2 converts extracellular ATP into 5’AMP which stimulates the expansion and accumulation of MDSCs in the tumors. Treatment of mice bearing Hepa1-6 tumors with ENTPD2 inhibitor POM-1 significantly inhibited HCC tumor growth and inhibited the accumulation of MDSCs. Corzo et al has reported that HIF1α induces differentiation of MDSCs into immunosuppressive TAMs. Hence, use of drugs that modulates expression, DNA-binding, transcription, or stabilization of HIF1α proteins can be used to decrease the recruitment of MDSCs in the TME (Fallah & Rini, 2019; Joshi et al., 2020; Joshi, Singh, & Durden, 2014; Joshi et al., 2014; Joshi, Singh, Zulcic, & Durden, 2014; K. X. Liu & Joshi, 2020; Noman et al., 2019; Tang & Zhao, 2020). TH-302 (evofosfamide), hypoxia-activated pro-drug has been used in various preclinical and clinical studies (Y. Li, Zhao, & Li, 2021). For instance, the combination of TH-302 with anti-CTLA 4 or PD1 blockade reduced hypoxia-induced accumulation of MDSCs as well as MDSC-mediated immunosuppression in mouse models of prostate cancer (Jayaprakash et al., 2018). This study leads to the opening of a clinical trial of TH-302 with ipilimumab in patients with prostate cancer, pancreatic cancer, HPV-head and neck cancer (NCT03098160). The studies are still ongoing, and no results are yet available. Recent single-cell RNA-seq studies on mouse tumor-infiltrating NK cells have shown that pharmacological inhibition of HIF 1α in NK cells reduced tumor growth and improved the anti-tumor activity of NK cells (Ni et al., 2020). In an extended culture of NK cells, KC7F2 (inhibitor of HIF1α translation) enhanced the cytotoxic function of NK cells. These emerging studies open the opportunities to combine HIF1α inhibitors with NK-cell targeted therapies to synergistically reduce MDSC accumulation and to enhance the cytotoxic potential of NK cells.

S100A8 and S100A9 are calcium-binding proteins that play a crucial role in the accumulation of MDSCs in the TME (Cheng et al., 2008; Gabrilovich, 2017; M. Huang et al., 2019; Sinha et al., 2008; Zhao et al., 2012). Inhibition of S100A8/A9 has been reported to reduce the accumulation of MDSCs in various mouse tumor models (Cheng et al., 2008; Sinha et al., 2008). Tasquinimod is in clinical development for the treatment of prostate cancer and other cancers and is identified to inhibit the binding of S100A9 proteins to TLR4 and RAGE receptors (L. Shen & Pili, 2019). Tasquinimod is reported to reduce infiltration and accumulation of MDSCs into TME, deplete blood monocytes, and promote polarization of TAMs into immunostimulatory M1 macrophages (Olsson et al., 2015; L. Shen & Pili, 2019; L. Shen et al., 2015). In Phase 2 clinical trial, administration of tasquinimod improved progression-free survival in patients with metastatic castration-resistant prostate cancer (mCRPC) by reducing the infiltration of MDSCs in the TME (Pili et al., 2011). In a phase 3 trial, tasquinimod treatment in patients with mCRPC, leads to prolonged progression-free survival compared to the placebo group (NCT01234311). In contrast to the results of these trials, a phase 2 clinical trial conducted on patients with advanced hepatocellular carcinoma, ovarian cancer, renal cell carcinoma, and gastric carcinoma showed no efficiency of tasquinimod on these tumors (NCT01743469) (Escudier et al., 2017). These studies suggest an important role of S100A8/A9 in promoting the accumulation of MDSCs, but further studies are needed to explore inhibitors of S100A8/A9 either alone or together with NK cell-based therapies.

Chemokine receptors are mainly responsible for the migration of MDSCs to the tumor site (Homey, Muller, & Zlotnik, 2002). MDSCs mainly express C-C motif chemokine receptor (CCR2) and are recruited in the tumors expressing chemokines, CCL2 and CCL5 (Lesokhin et al., 2012). Several studies have shown that blockade of the CCL2/CCR2 axis either alone or in combination with immunotherapy or targeted therapy decreased intra-tumoral MDSCs and improved anti-tumoral effects in various preclinical mouse models (Chang et al., 2016; Flores-Toro et al., 2020; Y. Wang, Zhang, Yang, Xue, & Hu, 2018). However, as a single agent, CNT0888, a humanized monoclonal antibody against CCL2, didn’t show any anti-tumor activity in mCRPC patients (Pienta et al., 2013). CCR2-targeting strategies have shown efficacy in cancer patients by reducing infiltration of monocytes in the tumors (Flores-Toro et al., 2020). PF-04136309 (CCR2 targeting agent) in combination with FOLFIRINOX has improved survival in patients with pancreatic adenocarcinoma (NCT01413022). The results have shown that patients treated with PF-04136309 and FOLFIRINOX combo showed improved anti-tumor responses due to reduced migration of monocytes from bone marrow and decreased generation of TAMs (Nywening et al., 2016). PF-04136309, in combination with gemcitabine and nab-paclitaxel was also evaluated in a Phase 2 clinical trial for patients with metastatic pancreatic ductal adenocarcinoma (NCT02732938). Another CCR2 inhibitor CCX872 has also been evaluated in combination with FOLFIRINOX in patients with pancreatic adenocarcinoma (NCT02345408). The combo group showed longer overall survival as compared to the monotherapy group. BMS-813160, is another CCR2 inhibitor that is currently tested in clinical trials in combination with immunotherapy (NCT03184870). Together, these studies have shown the efficacy of CCR2 targeting agents in reducing the frequency of MDSCs and improving the overall survival of cancer patients. However, these agents are not evaluated in combination with NK-cell-based immunotherapies, and hence preclinical studies are needed to study the combined efficacy of CCR2 targeting agents with adoptive transfer of NK-cells or cytokines like IL2 or IL-15 to demonstrate the efficacy before translating these studies into clinical trials.

CXCR1/2 signaling is upregulated in PMN-MDSCs and neutrophils. Hence targeting CXCR2 signaling either alone or together with anti-PD1 blocking antibody significantly improved T cell responses in both mice models and cancer patients (Izhak et al., 2010; Lesokhin et al., 2012). Several CXCR1/2 inhibitors like SX-682, reparixin, AZD-5069 have been evaluated in clinical trials for cancer patients and are listed in Table 1. CXCR2 inhibitor SX-682 has limited anti-tumor activity when used alone but together with checkpoint inhibitors, it shows great efficacy in improving anti-tumor immune responses (L. Sun et al., 2019). CXCR2 blockade by SX682 also enhanced the efficacy of NK cell therapy in head and neck cancers (Greene et al., 2020). SX682 inhibited MDSC accumulation and enhanced the therapeutic efficacy of adoptively transferred murine natural killer cells. These promising results suggest the therapeutic efficacy of this combination and open new avenues to explore CXCR2 inhibitors in combination with NK-cell adoptive transfer therapies in clinical trials.

The chemokine receptor CCR5 also plays an important role in the recruitment of MDSCs via ligands CCL3, CCL4 and CCL5. A study showed that blockade of CCR5 inhibited the recruitment of MDSCs and improved survival in melanoma (Blattner et al., 2018). Similarly, CCR5 blockade reduced tumor growth and metastatic potential in breast carcinoma, pancreatic, colorectal and prostate cancer (Balistreri et al., 2009; Combadiere, Ahuja, Tiffany, & Murphy, 1996; Robinson et al., 2003; Umansky, Blattner, Gebhardt, & Utikal, 2017). CCR5 antagonist, maraviroc is evaluated in combination with pembrolizumab in Phase I clinical trial for metastatic colorectal cancer (NCT03274804). In another phase I clinical trial, maraviroc is combined with ipilimumab and nivolumab to treat patients with metastatic colorectal and pancreatic adenocarcinoma (NCT04721301). These studies are still ongoing, and it’s too early to predict the efficacy of this drug in reducing MDSC accumulation and enhancing the anti-tumor immune responses nevertheless the strategy of altering chemokines to modulate cells in the TME is intriguing.

Targeting CSF1-R can also inhibit the recruitment of MDSCs to the tumor site. CSF1-R promotes the differentiation and expansion of myeloid cells when bound to ligand CSF1. CSF1R is upregulated in various cancers, including breast and pancreatic cancer (Holmgaard, Zamarin, Lesokhin, Merghoub, & Wolchok, 2016; Richardsen, Uglehus, Johnsen, & Busund, 2015; Y. Zhu et al., 2014). Preclinical studies in murine cancer models have shown that CSF1R inhibitors can block the increased secretion of various cytokines in TME, leading to decreased MDSC accumulation, angiogenesis, and tumor burden (Mao et al., 2016; Priceman et al., 2010; Webb et al., 2018). Targeting the CSF1/CSF1R axis either alone or in combination with checkpoint blockade, or adoptive T cell therapy has decreased tumor MDSCs and improved anti-tumor responses in multiple tumors (Mok et al., 2014; Sluijter et al., 2014). Various Phase 1 and phase 2 clinical trials are ongoing to evaluate the efficacy of CSF1-R inhibitors: Plexidartinib (NCT02452424), BLZ-945 (NCT02829723), and ARRY-382 (NCT02880371) with anti-PD1 mAbs (Table 1). No studies have been reported for CSF1-R inhibitors with NK cells and these studies are warranted in the near future.

### 9.2. Depletion of MDSCs

Chemotherapeutic agents have been shown effective in depleting MDSCs (Alizadeh & Larmonier, 2014; Draghiciu et al., 2015). Chemotherapy drugs, such as gemcitabine, 5-Fluorouracil, docetaxel, oxaliplatin, paclitaxel, and doxorubicin, exerts favorable effects by depleting MDSCs, increasing the efficacy of immune therapies, and enhancing the anti-tumor activity of NK cells (Alizadeh et al., 2014; Cao et al., 2015; Kodumudi et al., 2010; Michels et al., 2012; Sevko et al., 2013; Suzuki, Kapoor, Jassar, Kaiser, & Albelda, 2005; Vincent et al., 2010). Gemcitabine is reported to augment NK cell activation and enhance anti-tumor immune responses in a murine lung adenocarcinoma model by upregulating the expression of NKG2D ligand on lung cancer cells (X. Zhang et al., 2020). In another study, low dose gemcitabine inhibited the accumulation of MDSCs and increased the number of NK cells at the site of tumor resection in pancreatic carcinoma mouse model (Dawson & Fernandez-Zapico, 2016; Gurlevik et al., 2016). Meer *et al* have recently shown that CD34+ hematopoietic progenitor cell (HPC)-derived NK cells combined with gemcitabine decreased tumor growth in mice bearing ovarian cancer tumors (Van der Meer et al., 2021). Gemcitabine has not yet been explored in combination with NK cell therapies in human patients. However, compelling data generated on mouse models opened new avenues to use this combination strategy in clinical trials. Oxaliplatin is also reported to induce the expression of stress ligands and promote NK-cell mediated cytotoxicity in human ovarian cancer cells (Siew et al., 2015). The addition of activated NK cells completely suppressed the growth of oxaliplatin-treated cancer cells. In another study, the administration of 5-fluorouracil and oxaliplatin with adoptive transfer of autologous NK cells prevented recurrence and improved progression-free survival in patients with locally advanced colon carcinoma (Lingyu Li et al., 2017). Metformin, an anti-diabetic drug was also reported to reduce the frequency of circulating CD39^+^CD73^+^ MDSCs and increased the anti-tumor activity of T cells in patients with ovarian cancer (L. Li et al., 2018).

Signaling pathways involved in MDSC expansion have also been used as targets to reduce the populations of MDSCs. For example, tyrosine kinase inhibitors like sorafenib or sunitinib directly target VEGF and c-KIT signaling or inhibit MCSF or STAT3, which promote expansion of MDSCs (Ozao-Choy et al., 2009). Sunitinib was also found to inhibit STAT3, and treatment of sunitinib showed suppressed accumulation of MDSCs in renal cell carcinoma patients (Guislain et al., 2015; J. S. Ko et al., 2009; Xin et al., 2009). Sunitinib and sorafenib treatment also upregulates the expression of NKG2D and induces sensitivity of NK cells to tumor cells (Y. Huang, Wang, Li, Guo, & He, 2011; Y. X. Huang et al., 2017). Axitinib is reported to induce DNA damage in human renal carcinoma cells, which improved the killing of tumor cells by NK cells (Morelli et al., 2015). Ibrutinib, a tyrosine kinase inhibitor, is reported to deplete MDSCs in mice bearing EMT6 mammary tumors (Stiff et al., 2016) and neuroblastoma tumors (Ishfaq et al., 2021). Cabozantinib or celecoxib, a multi-targeted tyrosine kinase inhibitor, is recently reported to deplete MDSCs in mouse models of penile squamous cell carcinoma and castration-resistant prostate carcinoma (T. Huang et al., 2020; X. Lu et al., 2017). Cabozantinib, in combination with checkpoint inhibitor therapy, or cancer vaccine therapy eliminated MDSCs in murine models (T. Huang et al., 2020; X. Lu et al., 2017). Cabozantinib is also reported to synergize with EGFR specific CAR-NK-92 cells in human renal cell carcinoma xenograft models (Zhang et al., 2017). The study has shown that CAR-NK-92 cells lysed the renal cell carcinoma cells in an EGFR-specific manner. Thus, depleting MDSCs represents one important strategy to help normalize the TME and promote anti-tumor immunity. In addition, agents like gemcitabine and cabozantinib, which have shown great efficacy with NK-cell therapies in preclinical studies, should be evaluated in clinical trials.

### 9.3. Inducing MDSC Differentiation

Inducing the differentiation of immature myeloid cells is also used to reduce the number of MDSCs in murine tumor models and cancer patients. Several studies have shown that vitamin A, D3, and E decrease immature MDSCs and enhance the anti-tumor activity of T cells in murine models and head and neck cancer patients (Lathers, Clark, Achille, & Young, 2004; Wiers, Lathers, Wright, & Young, 2000). One study has shown that patients deficient in vitamin D have lower NK-mediated cytotoxicity (Mortara et al., 2018). Vitamin E enhances immune responses by reducing ROS levels and NO production (G. Y. Lee & Han, 2018). MDSCs inhibit the function of NK cells via NO production; hence using vitamin E can target MDSC-NK interaction and crosstalk. In addition, casein kinase inhibitors enhance myeloid cell differentiation in mice bearing tumors and synergize with checkpoint inhibitor CTLA4 (Hashimoto et al., 2018).

All trans-retinoic acid (ATRA), a metabolite of Vitamin A, skews the differentiation of MDSCs into mature myeloid cells (Y. Li, Wongsiriroj, & Blaner, 2014; Mirza et al., 2006; Nefedova et al., 2007). ATRA treatment has resulted in the differentiation of mature antigen-presenting precursor cells leading to suppression of T cell responses in both mice models and various human cancers (Iclozan, Antonia, Chiappori, Chen, & Gabrilovich, 2013; A. H. Long et al., 2016; Mirza et al., 2006). In phase 2 clinical trial, ATRA either alone or combined with DC vaccine against p53 showed promising results in reducing frequencies of MDSCs in patients with small-cell lung cancer (NCT00617409) (Iclozan et al., 2013; Mirza et al., 2006). The combined treatment decreased MDSCs and enhanced granzyme-positive CD8+T cell responses in patients. In another study, ATRA treatment reduced the number of MDSCs and improved the efficacy of CAR therapy in sarcoma models (A. H. Long et al., 2016). In advanced-stage melanoma patients, ATRA alone or in combination with ipilimumab is reported to reduce the circulating levels of MDSCs (Tobin et al., 2018). ATRA treatment also improved the efficacy of anti-angiogenic therapy in a preclinical breast cancer model (R. Bauer et al., 2018). ATRA is reported to modulate the expression of MICAA/B, leading to increased activation of NK cells and enhanced production of IFN gamma by NK cells co-cultured with ATRA-treated hepatoma cells (Jinushi et al., 2003; Nwangwu, Weiher, & Schmidt-Wolf, 2017). ATRA is also reported to upregulate chemerin to promote NK cell recruitment in melanoma tumors (Song et al., 2019). Based on the exciting clinical data demonstrating reduced circulating levels of MDSCs in patients treated with ATRA and recent studies illustrating that ATRA can enhance the activation of NK cells, clinical trials combining ATRA with NK-cell based therapies is warranted in near future.

### 9.4. Blocking Immunosuppressive functions of MDSC

Modulating immunosuppressive mechanisms employed by MDSCs to shut off immune responses have also been used as a therapeutic strategy to increase the cytotoxic activity of T and NK cells. As discussed previously the STAT family of transcription factors, especially STAT3, plays a major role in the accumulation and expansion of MDSCs in tumors (Condamine et al., 2015; Nefedova et al., 2004). Hence, pharmacological inhibition of STAT3 by small molecule inhibitors or curcumin inhibitors blocks suppressive functions of MDSCs in various preclinical mouse models (L. Lin et al., 2010; J. F. Liu et al., 2018; P. Lu, Yu, & Xu, 2012). JAK/STAT3 inhibitors also reduce MDSC trafficking in tumors by inhibiting VEGFA (J. F. Liu et al., 2018). Some studies have shown that inhibition of STAT3 increases the expression of NKG2D, resulting in enhanced NK cell cytotoxicity (Sui et al., 2014; X. Sun et al., 2013). Other approaches using STAT3 oligonucleotide decoy inhibitor, AZD9150 (Danvatirsen), or STAT3 small interfering RNA (siRNA) either alone or together with checkpoint inhibitors decreased granulocytic MDSCs in preclinical mouse models as well as in Phase I/II clinical trials (Kortylewski & Moreira, 2017; Spinetti et al., 2016; Q. Zhang et al., 2016). Danvatirsen monotherapy showed durable clinical responses in two clinical trials (NCT01563302 and NCT01839604). In a phase 1b clinical trial, danvatirsen treatment reduced levels of PMN-MDSCs in patients with diffuse B cell lymphoma (NCT01563302) (Reilley et al., 2018). Danvatirsen when combined with durvalumab (anti-PDL1 mAb) has shown clinical benefit in patients with advanced solid tumors (NCT02983578). The preclinical studies done on the CT26 murine colorectal model using both danvatirsen and durvalumab showed a decreased frequency of M-MDSC and G-MDSCs and an increase in the number of NK cells and T cells in the treated tumors (Proia et al., 2020). The combined treated group showed an increase in the expression of granzyme in NK cells, leading to enhanced NK cell cytotoxicity. In another clinical trial, patients receiving a combination of durvalumab and danvatirsen showed enhanced efficacy as compared to duravulumab monotherapy or duravulumab and AZD5069 (CXCR2 inhibitor) treated group (NCT02499328) (Cohen et al., 2018). TLR7 pathway activation also plays a role in MDSC differentiation, and this activation is mainly mediated through signaling via JAK/STAT pathway (Larange, Antonios, Pallardy, & Kerdine-Romer, 2009). Hence, TLR7/8 agonist resiquimoid (R848) is reported to reduce both intratumoral and circulating MDSCs as well as block the immunosuppressive function of MDSCs in CT26 colon carcinoma model (Spinetti et al., 2016). In another study, oxaliplatin combined with resiquimoid reversed oxaliplatin resistance in colorectal cancer by inducing polarization of MDSCs into M1-like macrophages (Z. Liu et al., 2020). Recently, combinatorial inhibition of the STAT3 pathway and activation of TLR7/8 pathway have been reported to suppress the activity of MDSCs in patients with breast cancer (Safarzadeh et al., 2020).

Histone deacetylase (HDAC) inhibitors also play an important role in reducing MDSC mediated immunosuppression and enhancing NK cell cytotoxicity (Cui, Cai, Wang, & Wang, 2021; H. F. Wang et al., 2017). For instance, HDAC 1 inhibitor, entinostat, has also been reported to improve NK cell responses by reducing the immunosuppressive functions of MDSCs (Kim et al., 2014; Orillion et al., 2017). These studies also observed a substantial reduction in tumor-infiltrating macrophages, suggesting the predominant effect of this drug on myeloid cells. Entinostat is combined with nivolumab and azacytidine in phase 2 clinical trial of patients with non-small lung cancer (NCT01928576). Entinostat also enhanced NK cell-mediated cytotoxicity by increasing expression of NK ligands (Hicks et al., 2018). Kiany et al have shown that entinostat has upregulated ligands for NK cell receptors MICA and MICB on osteosarcoma cells both *in vitro* and *in vivo* and enhances NK-cell mediated cytotoxicity *in vitro* (Kiany, Huang, & Kleinerman, 2017). However, entinostat failed to augment the efficacy of NK cell therapy (*in vitro* expanded NK cells) in nude mouse human osteosarcoma lung metastasis model. There can be many factors that can lead to decreased efficacy of this combination, and future studies are required using combination of entinostat with different NK cell therapies like adding NK-cell-activating cytokine IL2 or IL-15. Zhu et al have shown that entinostat treatment increased the expression of NKG2D on primary human NK cells to enhance NK cell-dependent recognition of cancer cells. Together, these studies indicate that entinostat can enhance NK-cell targeted therapies for solid tumors (S. Zhu et al., 2015). HDAC 6 inhibitor, ricolinostat reduced M-MDSCs but didn’t reduce PMN-MDSCs and was not able to reduce tumor growth in EL4 and LLC tumor models (Hashimoto, Fukumoto, Zhang, & Gabrilovich, 2020). However, in combination with entinostat, it completely abrogated both populations of MDSCs and delayed tumor progression. Another HDAC inhibitor, valproic acid is also reported to block functions of MDSCs and enhance anti-PDL1 immunotherapy (Xie, Ago, Okada, & Tachibana, 2018). Xie et al has shown that valproic acid limits CCR2 dependent infiltration of m-MDSCs into EL-4 and B16 tumor models and, in combination with anti-PD1 enhanced anti-tumor immune responses (Xie, Ikegami, Ago, Okada, & Tachibana, 2020). The study has also shown that valproic acid relieved MDSC-mediated immunosuppression in the tumors and enhanced the proliferation of CD8+T cells and NK cells.

ARG-1 small molecule inhibitors are reported to decrease iNOS and COX-2 levels and modulate immunosuppressive functions of MDSCs (Rodriguez et al., 2004; Steggerda et al., 2017). A study by Steggerda et al. has shown that small peptide inhibitor of ARG-1 combined with anti-PDL1 reduced tumor growth (Steggerda et al., 2017). ARG-1 inhibitor (CB-1158) decreased MDSC recruitment in TME, increased tumor-infiltrating T cells and NK cells, reduced tumor burden in preclinical models (Steggerda et al., 2017). This study further demonstrated that CB1158 in combination with the adoptive transfer of NK cells reduced tumor growth and metastasis in CT-26 colorectal adenocarcinoma model. In a phase I clinical trial, CB1158 combined with pembrolizumab was well tolerated in patients with advanced and metastatic tumors (NCT02903914). Based on exciting phase I clinical data and preclinical data, CB1158 can be explored with the adoptive transfer of NK cells to treat advanced cancers.

Moreover, several preclinical and clinical studies show that phosphodiesterase-5 (PDE5) inhibitors sildenafil and tadalafil decrease MDSC accumulation and pro-tumor functions of MDSC in the TME (Califano et al., 2015; Hamilton et al., 2013; Hassel et al., 2017; S. Lin et al., 2017; Serafini et al., 2006; Tai et al., 2018; Weed et al., 2015). These inhibitors reduced MDSC function through downregulation of ARG, and iNOS expression. In an open-label phase trial, tadalafil treatment was well tolerated in patients with metastatic melanoma. In addition, the patients with stable disease show reduced infiltration of MDSCs in tumor lesions (Hassel et al., 2017). Tadalafil has inhibited the function of MDSCs and improved anti-tumor immune response in patients with head and neck squamous cell carcinoma (NCT01697800) (Califano et al., 2015; Tai et al., 2018; Weed et al., 2015). In another Phase 1/2 clinical trial, tadalafil is combined with anti-tumor mucin 1 (MUC-1) vaccine to reduce the number of MDSCs and T regs in patients with head and neck squamous cell carcinoma (Weed et al., 2019). Together, these studies have shown that PDE5 inhibitors increased NK cell cytotoxicity and enhanced CD8+ T cell function in the TME. COX2 inhibitor, celecoxib is also reported to reduce immunosuppressive actions of MDSCs in preclinical studies (Iachininoto et al., 2013; Kosaka, Ohkuri, & Okada, 2014; Veltman et al., 2010).

Inhibition of TGFβ signaling has also shown promising results in reducing MDSC-mediated immunosuppression in preclinical models and clinical studies (Fujiwara et al., 2015; Shaim et al., 2021; Tran et al., 2017; Yingling et al., 2018). Therapies aimed to target TGFβ signaling have improved NKG2D-mediated tumor recognition and enhanced tumor cell lysis by NK cells and T cells (Y. S. Lee et al., 2021; Otegbeye et al., 2018; Tran et al., 2017). Galunisertib (LY2157299) is a small molecule that inhibits the kinase activity of TGFβR1 and has been evaluated in various clinical trials (NCT01722825 and NCT00356460). Otegbeye et al have shown that administration of galunisertib increased the anti-tumor effect of adoptively transferred NK cells in a mouse liver metastases model of colon carcinoma (Otegbeye et al., 2018). TGFβ specific mAbs are also evaluated in phase I and phase II clinical trials for solid tumors (Batlle & Massague, 2019). TGFβ inhibitors have so far shown significant efficacy in improving the cytotoxicity, and anti-tumor functions of NK cells, and future clinical trials combining TGFβ inhibitors with NK cell therapies are warranted.

## 10. Conclusions

The establishment of an immunosuppressive TME is linked to tumor progression and MDSCs play a central role in this process. MDSCs are known to interact with adaptive immune cells and suppress their function to promote tolerance in cancer. As such, targeting MDSCs has received significant attention in combination with T-cell targeted therapies. However, the interaction between MDSCs and innate immune cells, in particular NK cells is less understood. Here we highlighted and summarized known mechanisms by which MDSC can modulate NK cells and current strategies targeting MDSCs to improve NK cell anti-tumor activity. Understanding the diverse interactions between MDSC and both adaptive and innate effector cells, including NK cells, is critical to designing combinatorial strategies to overcome immunosuppression and unleash a robust and multifaceted anti-tumor immune response.

## Figures and Tables

**Table 1:** Strategies for targeting MDSCs. Table includes ongoing clinical trials to target MDSCs in cancer patients. It also includes preclinical studies done on mouse models to evaluate the efficacy of MDSC targeted agents with NK-cell therapies.

Strategy	Combination	Cancer Type	Clinical trial	Outcomes
**Targeting MDSC recruitment**				
Bevacizumab		NSCLC		Reduced PMN-MDSCs in peripheral blood of patients treated with bevacizumab
	EGFR tyrosine kinase inhibitor	EGFR mutant lung adenocarcinoma		Reduced S100A-9 positive MDSCs in peripheral blood of patients with lung adenocarcinoma and improved progression free survival
	Capecitabine	Recurrent glioblastoma	NCT02669173	Reduced circulating MDSCs in patients
	5-Fluorouracil and oxaliplatin	Colorectal cancer		Reduced circulating MDSCs in patients
	Umbilical-cord derived NK cells	Human LoVo colorectal tumors		Decreased accumulation ofMDSCs and MDSC-mediated immunosuppression
	Allogenic NK cells	Metastatic solid tumors	NCT02857920	No results available
TH-302	Anti-CTLA-4/anti-PD1	Mouse model of Prostate cancer		inhibited MDSC accumulation, activation of NK cells in mouse model
	Ipilimumab	Metastatic prostate cancer, pancreatic cancer, HPV-head, and neck cancer	NCT03098160	No results posted
Tasquinimod		Metastatic castration-resistant Prostate cancer	NCT01234311	Improved progression-free survival by inhibiting MDSC accumulation
		advanced hepatocellular carcinoma, ovarian cancer, renal cell carcinoma, and gastric carcinoma	NCT01743469	No efficiency in improving survival
PF-04136309	FOLFIRINOX	Locally advanced pancreatic adenocarcinoma	NCT01413022	Improved survival, reduced migration of monocytes from bone marrow
	Gemcitabine and nab-paclitaxel	Metastatic pancreatic adenocarcinoma	NCT02732938	Improved survival in combo treated group
CCX872	FOLFIRINOX	pancreatic adenocarcinoma	NCT02345408	Improved overall survival
BMS-813160	Nivolumab and chemotherapy	advanced solid tumors	NCT03184870	No results posted
SX682	Adoptively transferred murine NK cells	Murine model of Head and neck cancer		Enhanced anti-tumor immune responses and therapeutic efficacy of adoptively transferred NK cells
	Pembrolizumab	Metastatic melanoma	NCT03161431	No results posted
	Nivolumab	Metastatic pancreatic ductal adenocarcinoma	NCT04477343	No results posted
Reparixin	Paclitaxel	Metastatic triple negative breast cancer	NCT02370238	No results posted
AZD5069	Enzalutamide	Metastatic castration-resistant Prostate cancer	NCT03177187	No results posted
Maraviroc	Pembrolizumab	Metastatic colorectal carcinoma	NCT03274804	Clinical activity is limited, but prolonged disease stabilization was observed
Plexidartinib	Ipilimumab and nivolumab	Metastatic colorectal and pancreatic carcinoma	NCT04721301	No results posted
	Pembrolizumab	Melanoma	NCT02452424	Insufficient clinical efficacy
BLZ-945	PDR001	Solid tumors	NCT02829723	No results posted
ARRY-382	Pembrolizumab	Solid tumors, NSCLC, melanoma	NCT02880371	No results posted
**MDSC depletion**				
Gemcitabine	CD34+ HPC-NK cells	Murine model of ovarian cancer		Decreased tumor growth
5-fluorouracil	Oxaliplatin +NK cells	advanced colon carcinoma		prevented recurrence and improved progression-free survival
Cabozantinib	EGFR-specific CAR-NK92 cells	Human renal cell xenograft studies		CAR-NK-92 cells lysed the renal cell carcinoma cells in an EGFR-specific manner improved the killing of tumor cells by NK cells
**Inducing MDSC differentiation**				
ATRA	DC vaccine against p53	Small cell lung cancer	NCT00617409	Reduced frequency of MDSCs in patients with lung cancer
	ipilimumab	Melanoma	NCT02403778	Reduced circulating MDSCs
**Blocking Immunosuppressive functions of MDSC**				
AZD9150(Danvatirsen)		Diffuse large B cell lymphoma	NCT01563302	Reduced levels of PMN-MDSCs
		Metastatic hepatocellular carcinoma	NCT01839604	One patient in the escalation cohort showed partial response
	duravulumab	Advanced solid tumors	NCT02983578	No results posted
	duravulumab AZD5069	Advanced solid tumors and HNSCC	NCT02499328	Danvatirsen plus duravulumab showed enhanced activity as compared to duravulumab and AZD5069 or duravulumab alone
Entinostat	Nivolumab Azacytidine	Non-small cell lung cancer	NCT01928576	No results posted
		Nude mouse osteosarcoma metastasis model		Failed to augment the efficacy of NK cell therapy
CB-1158	Infused NK cells pembrolizumab adoptive transfer of NK cells	Advanced and metastatic tumors	NCT02903914	Well tolerated in patients, increase in plasma arginine levels
		Murine model of colorectal carcinoma		Reduced tumor growth and metastasis
Tadalafil		head and neck squamous cell carcinoma	NCT01697800	Inhibited MDSC function and improved anti-tumor immune responses.
	Anti-tumor Mucin-1 vaccine	head and neck squamous cell carcinoma	NCT02544880	Reduced number of MDSCs and Tregs in treated patients
Galunisertib	Adoptively transferred NK cells	mouse model of colon carcinoma		Increased the anti-tumor effect of adoptively transferred NK cells

## Abbreviations:

TME: tumor microenvironment
MDSC: myeloid-derived suppressor cells
M-MDSCs: monocytic MDSCs
PMN-MDSCs or G-MDSCs: polymorphonuclear or granulocytic MDSCs
eMDSC: early-stage MDSC
STAT: signal transducer and activation of transcription
Treg: regulatory T cells
TAM: tumor-associated macrophages
TAN: tumor-associated neutrophils
CMP: common myeloid progenitors
MCSF: macrophage colony-stimulating factor
LOX-1: lectin-type oxidized LDL receptor-1
HPC: hematopoietic progenitor cells
GMP: granulocyte-macrophage progenitors
IMC: immature myeloid cells
MB: myeloblasts
BM: bone marrow
NK: natural killer cells
PGE2: prostaglandin E2
GMCSF: granulocyte-macrophage stimulating factor
GCSF: granulocyte-colony stimulating factor
VEGF: vascular endothelial growth factor
TGFβ: transforming growth factor
MHC: major histocompatibility complex
CAR: Chimeric antigen receptors
mCRPC: metastatic castration-resistant prostate cancer
IL-10: interleukin 10
NCR: natural cytotoxic receptor
PGE2: Prostaglandin E2
IDO: indoleamine 2, 3 dioxygenase
ADCC: antibody-dependent cellular cytotoxicity
ROS: reactive oxygen species
MMPs: matrix metalloproteinase
